# PorA Variable Regions of *Neisseria meningitidis*

**DOI:** 10.3201/eid1004.030247

**Published:** 2004-04

**Authors:** Joanne E. Russell, Keith A. Jolley, Ian M. Feavers, Martin C. J. Maiden, Janet Suker

**Affiliations:** * University of Oxford, Oxford, United Kingdom; † National Institute for Biological Standards and Control, South Mimms Potters Bar, United Kingdom

**Keywords:** *Neisseria meningitidis*, PorA protein, nomenclature, subtyping, worldwide web, vaccine, monoclonal antibody, antigenic variation

## Abstract

Subtypes, defined by variation in the outer membrane protein PorA, are an integral part of the characterization scheme for *Neisseria meningitidis*. Identification of these variants remains important as the PorA protein is a major immunogenic component of several meningococcal vaccines under development, and characteristics of PorA are used to provide detailed epidemiologic information. Historically, serosubtypes have been defined by reactivity with a set of monoclonal antibodies. However, nucleotide sequence analyses of *porA* genes have established that the panel of serosubtyping monoclonal antibodies is not exhaustive and many *porA* variants cannot be detected. In addition, the nomenclature system used to define subtypes is inadequate. We examined all available nucleotide sequences of the *porA* VR1 and VR2 regions to identify and define subtype families. A revised nomenclature scheme, compatible with the previous serologic nomenclature scheme, was devised. A Web-accessible database describing this nomenclature and its relationship to previous schemes was established (available from: http://neisseria.org/nm/typing/pora).

*Neisseria meningitidis* is a major cause of bacterial meningitis and septicemia worldwide (*1*). In the absence of a comprehensive vaccine against this organism, the characterization of its variable surface antigens is important for epidemiologic monitoring and vaccine development (*2*). The serologic characterization scheme for meningococci comprises the following: groups, based on variants in the capsular polysaccharide; types, based on variants of the PorB outer membrane protein (OMP); subtypes, based on variants of the PorA OMP; and immunotypes, based on variants in the lipooligosaccharide (*3*). Within this scheme, PorA, also known as the class 1 OMP, is assigned the prefix “P1.” followed by numbers, separated by commas, that correspond to the subtype designation (thus: P1.7,16). The two PorA variable regions (VR1 and VR2) that confer the subtypes are especially important because they elicit bactericidal antibodies in humans (*4*). Consequently, a number of meningococcal vaccines under development contain the PorA protein as a major component (*5*).

Nucleotide sequence analyses of *porA* genes from multiple meningococcal isolates have established that the panel of serosubtyping monoclonal antibodies (MAbs) is not comprehensive. Meningococci are frequently only partially serosubtyped, and an increasing number of isolates are classified as non-serosubtypeable, either because a variant is not recognized by MAbs or because PorA is not expressed. This heterogeneous group of isolates can be fully characterized on the basis of their PorA VR1 and VR2 amino acid sequences deduced from nucleotide sequence data. To accommodate subtypes identified on the basis of sequence data alone, the scheme originally developed for MAb reactivity data (*3*) was modified so that VR families and variants were assigned on the basis of amino acid sequence relationships rather than their reactivity with specific MAbs. A distance matrix of all known VR1 and VR2 amino acid sequences was constructed, and VR amino acid sequences containing >80% identity to each other were grouped into VR families. The VR epitope recognized by an existing MAb raised against PorA, or the first defined amino acid sequence of a VR family, was arbitrarily designated as the prototype VR for that particular family. Successive distinct members of a VR family were designated as minor variants of that family, and as such were sequentially assigned an additional unique lower case letter, e.g., P1.5a, P1.5b, P1.5c (*6*).

Although this nomenclature system was sufficiently flexible to accommodate both novel subtypes determined from nucleotide sequence analyses and those defined by the reactivity of specific MAbs, limitations have become apparent. First, while the 80% similarity rule has generally proved adequate to assign VR families, it is open to misinterpretation, leading to the inappropriate designation of VR sequences. Second, the assignment of minor variants within VR families is limited by the number of letters in the alphabet (*7*,*8*). We present a revised nomenclature, which addresses these issues and shows the relationship of new designations to the previous designations and to the reactivities of the MAb panel. A database accessible through the Internet has been established, which will enable this scheme to be continually updated.

## Materials and Methods

### Bacterial Isolates

Two sets of meningococcal isolates were used for *porA* gene sequencing in this work. The first was a set of 393 isolates from cases of disease from diverse locations throughout the United Kingdom. These included 125 isolates from 1975; 100 isolates from 1985; 100 isolates from1995; and 18 urethral isolates, provided by the Meningococcal Reference Unit, Manchester Public Health Laboratory, Manchester. Fifty isolates were provided by the Scottish Meningococcus and Pneumococcus Reference Laboratory, Glasgow. The second set of isolates included the 107 globally representative isolates obtained from both patients and carriers; these isolates were used to develop and evaluate the multilocus sequence typing isolate characterization scheme (*9*).

### *porA* Gene Sequences and Validation

Nucleotide sequences of *porA* genes encoding the variants included in Appendix Tables 1 and 2 were obtained from the literature or GenBank, determined by sequencing of polymerase chain reaction (PCR) products from the above isolates, or submitted by personal communication or to the PorA Web site. Where possible, sequences not determined in this study were validated by requesting sequence electropherograms from depositors. When electropherograms could not be resolved, isolates were requested and the *porA* genes resequenced. Seven sequences contained errors on resequencing original isolates and were therefore removed from the new nomenclature scheme. The deposited VR sequences used in this study were those submitted to the PorA Web site by June 11, 2001.

### DNA Amplification and Nucleotide Sequence Determination of *porA*

Boiled meningococcal suspensions or DNA prepared from such suspensions with an Isoquick kit (Microprobe Corporation, Washington) were used as template to amplify the *porA* gene by using Taq Polymerase (Applied Biosystems, by Roche Molecular Systems Inc., Branchburg, NJ) with primers 210 and 211 (*10*). The amplification products were purified by precipitation with the addition of 0.6 V of 20% polyethylene glycol 8000/2.5M NaCl (*11*) and their nucleotide sequences determined at least once on each DNA strand. Sequence reactions were carried out with primers 8L, 8U, 103L, 103U, 122L, 122U, 210, and 211 (*10*) using BigDye Ready Reaction Mix (Applied Biosystems) in accordance with the manufacturer’s instructions. Unincorporated dye terminators were removed by precipitation of the termination products with the addition of 2.6 V of 96% ethanol and 115 mM sodium acetate. The reaction products were then separated and detected with an ABI Prism 377 automated DNA sequencer (PE Biosystems). Sequences were assembled from the resultant electropherograms with the STADEN suite of computer programs (*12*).

### Manipulation and Alignment of Sequences

Sequences were manipulated in SeqLab, part of the GCG software package (*13*). All unique nucleotide sequences for each VR were aligned with reference to both the nucleotide and the amino acid sequences, such that all sequences remained in frame, gaps were minimized, and similar codons were aligned.

### Identification of Families and Variants

To remain consistent with serologic and historical nomenclature, where a variant had been identified previously by serologic means, the identified sequence was used as a family prototype around which new sequences were grouped. An 80% amino acid identity cut-off— against the shortest sequence length when the sequences were of different length to allow for insertions, duplications, and deletions—was used as a guide in this grouping. In a few cases, a variant was assigned to a particular family even though the amino acid identity was slightly less than 80%, compared to the family prototype. In these cases, the new variant was still more similar to this particular family than to others but also contained a particular motif that was representative of family members. Therefore, a combination of overall similarity and presence of particular motifs was used to make the groupings. In a few cases, family-specific motifs were missing, but the sequences were otherwise identical or highly similar to members of the family. In such cases, the sequence was assigned as a variant of the family.

To further ensure that family groupings were consistent, the relationships among aligned nucleotide sequences encoding VR1 or VR2 were visualized by split decomposition analysis using SPLITSTREE version 3.1 (*14*). The split decomposition analysis was carried out in a sequential manner. In each analysis, a limited number of families were resolved, and the remaining variants were clustered together at a node. The variants that were resolved first were removed, and the analysis was repeated to resolve further families, and so on until all family groups were resolved (*15*). For analysis of the whole datasets, Hamming distances (equivalent to *p*-distance) were used because some of the families were so diverse that using a substitution model was not possible. This method resolved the most distantly related families. The Kimura three parameter model (*16*) was used to determine whether related sequences constituted families.

A database and Web site containing all the assignments have been established (available from: http://neisseria.org/nm/typing/pora). The sequences are stored in a PostgreSQL database running on Linux. Perl scripts enable the database to be queried against either peptide or nucleotide sequences; when an identical match is not found, a BLAST search (*17*) can be performed to identify the nearest variant and family. Any length of sequence can be queried, enabling the variants to be quickly identified from a whole or partial gene sequence.

## Results

### Validation of Sequence Variants

The sequences defining the following subtypes in the previous nomenclature were not included in the new nomenclature as a consequence of the sequence validation: P1.2a, P1.2d, P1.5b, P1.10h, P1.10i, P1.10j, P1.18b, P1.19c, P1.24, P1.29.

### Resolution of VR Families

**Figure 1:**
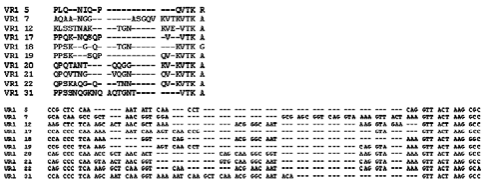
Alignment of the amino acid and corresponding nucleotide sequence of each VR1 family “prototype.”

**Figure 2:**
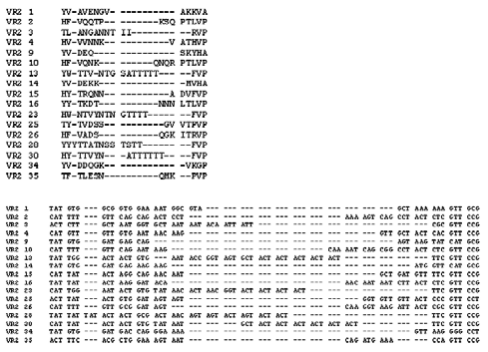
Alignment of the amino acid and corresponding nucleotide sequence of each VR2 family “prototype.”

The amino acid sequences of the prototype member of each of the VR families identified are shown aligned in Figures 1 and 2 together with corresponding nucleotide sequences. A total of 10 VR1 and 17 VR2 families were resolved. The most closely related VR families are VR2 P1.2 and VR2 P1.10, although the family prototypes are recognized by specific MAbs that are not cross-reactive. Both families start with a consensus amino acid sequence of HFVQ and end with PTLVP. They can be differentiated, however, by split decomposition where they cluster separately (*15*) and by certain motifs in their sequences. The P1.10 family members have a consensus motif QNKQNQ, with either the first or second triplet commonly repeated, while the P1.2 family members usually start with HFVQQ and commonly have variations of PQSQ or PKSQ. Grouped within the P1.2 family are four sequences that were previously designated as the P1.33 family. Like sequences in most of the P1.2 family members, these start with HFVQQ and, although they mostly end with SKPTLVP rather than SQPTLVP, they maintain the position of the serine residue.

### Variation within Families

There was more variation within VR2 (161 unique variants) than in VR1 (73 unique variants). The variation in the VR families was mainly due to changes that could be ascribed to single nonsynonymous base changes. Although there may be minor differences in the relative contribution of nonsynonymous base changes and insertions or deletions between individual VR families, approximately twice as many variants have arisen as a result of point mutations than from any other type of mutation. The repetition of amino acid motifs or single residues was common within VR2. An example is the repetition of a threonine residue within the VR2 P1.13 family, where there are sequences with three to nine consecutive threonine residues.

### Nomenclature Scheme

A consultation process was conducted by email among users of the PorA Web site and other interested parties. Several formats for a revised nomenclature were proposed and a request for alternatives made. The consensus opinion was to replace letters with numbers in subtype variant names in the following format: the prefix “P1.” followed by the VR1 family name, followed by a dash and then the variant number, followed by a comma and the VR2 variant name in the same format. When a family prototype VR, or first sequence belonging to a family, was identified, no variant number was used; for example, a protein with VR1 family 5 variant 3, and VR2 family 10 prototype would be written as: P1.5‑3,10. This scheme was then used to rename all of the variants examined. The new names of variants are listed in Appendix Tables 1 and 2, together with the previous nomenclature, peptide sequence, and source or reference. A database accessible through the Internet was established (available from: http://neisseria.org/nm/typing/pora).

## Discussion

These analyses confirm that, while diverse, the VR1 and VR2 peptide sequences can be assigned to distinct meningococcal PorA variant sequence families. However, these regions of the PorA protein are likely to be exposed to continual selection imposed by host immune responses, and VR families might evolve over time into different families. The similarity of the P1.2 and P1.10 VR2 families is perhaps a consequence of relatively recent divergence of one VR family into two. Devising a scheme for defining the boundaries of VR families that accurately reflects the evolution of these regions is therefore not possible. Moreover, the high diversity of these sequences presents problems in developing a facile nomenclature. In revising the nomenclature system, we used amino acid sequences, deduced from nucleotide sequences, of the two VRs as the definition of subtype variants. The replacement of letters with numbers in subtype variant designations overcomes the shortage of letters but entails a change of name of variants.

Since MAbs are still routinely used globally for meningococcal serosubtyping, to avoid confusion, family names from the previous nomenclature were retained when possible, and especially when the family prototype was specifically recognized by a typing MAb. The new nomenclature builds on the previous designations but has the advantage of a limitless capacity for expanding the number of variants included. Retaining family names, when they can be shown to be reasonable, results in some minor changes to some family groupings. As meningococci evolve, the use of nucleotide sequencing to determine the VR peptide sequences will be increasingly important for epidemiologic studies and vaccine design, especially as the MAb panel gradually becomes less useful and sequencing technology becomes more available.

In the course of this study, a number of VR sequences that had been deposited previously in GenBank were found, when resequenced, to contain errors and were in fact previously identified variants. These sequences had been given new variant names and, in two cases, were sufficiently novel to warrant the naming of new families. The widespread use and, more importantly, the comparison of VR sequence data among different laboratories require consistency of nomenclature and a high level of data accuracy. One way to achieve this is through a central PorA database in which sequence electropherograms are submitted for verification before new variant numbers are assigned. We have established a Web site for this role (available from: http://neisseria.org/nm/typing/pora). All known variants are listed, and a database query page is provided so that a VR sequence can be typed or pasted in and identified if previously seen. The Web site also includes links to the porB typing and MLST Web pages. The PorA Web site is now in widespread use by the research community and provides a single point of focus to ensure consistency in identifying and naming this important protein.

## Supplementary Material

Appendix Table 1 VR1 sequence nomenclaturea

Appendix Table 2 VR2 sequence nomenclaturea
