# Supplementary material for: PorA Variable Regions of Neisseria meningitidis
Source: Emerg Infect Dis. 2004 Apr;10(4):674–8. doi: 10.3201/eid1004.030247 (PMC3323080; doi:10.3201/eid1004.030247)
Supplement: Appendix Table 1 — VR1 sequence nomenclaturea [file 03-0247-appT1-s1.pdf]

Appendix Table 1. VR1 sequence nomenclature[a]

Variant	Previous nomenclature	VR1 peptide sequence	Source or reference
5	5	PLQNIQPQVTKR	(1)
5–1	5a	PLQNIQQPQVTKR	(2,3)
Removed	5b	PALPNIQPQVTKA	(4)
5–2	5c	PLPNIQPQVTKR	(3)
5–3	5d	PLQNIKQPQVTKR	(5)
5–4	5e	PLQNIQKPQVTKR	(6)
5–5	-	PLQNIQPSVTKR	WS
5–6	-	LLQNIQQPQVTKR	WS
7	7	AQAANGGASGQVKVTKVTKA	(7)
7–1	7a	AQAANGGAGASGQVKVTKVTKA	(1)
7–2	7b	AQAANGGASGQVKVTKA	(3)
7–3	7c	AQAANGGARASGQVKVTKVTKA	(3)
7–4	7d	AQAANGGAGASGQVKVTKA	(3)
7–5	7e	AQAANGGAVASGQVKVTKVTKA	(5)
7–6	7f	AQAANGGASDQVKVTKA	GenBank AF146084
7–7	7g	AQSANGGASGQVKVTKVTKA	(6)
7–8	7h	AQAANGGAGASGQVKVTKVTKVTKA	(6)
7–9	7i	AQAANGGASGANGGASGQVKVTKA	This study
7–10	-	AQAANGGVSGQVKVTKVTKA	WS
7–11	-	AQAANGGASGQVKVTKVTKVTKA	WS
12	12	KLSSTNAKTGNKVEVTKA	(1)
12–1	12a	KPSSTNAKTGNKVEVTKA	(8)
12–2	12b	KPSSTKAKTGNKVEVTKA	(5)
12–3	12c	KPSSTNAKTGNKVKVTKA	WS
12–4	12d	KSSNTNAKTSNKVEVTKA	WS
12–5	12e	KPSSTNPKTGNKVEVTKA	(6)
12–6	12f	QPSNTNGKTGNKVEVTKA	(6)
12–7	-	KPSSTNANSSTNAKTGNKVEVTKA	WS
12–8	-	KPSSTNAKTSNEVEVTKA	WS
17	17	PPQKNQSQPVVTKA	(1)
17–1	17a	PPPKNQSQPVVTKA	This study
17–2	-	PPQKNQSQPLVTKA	WS
18	18	PPSKGQTGNKVTKG	(1)
18–1	18a	PPSQGQTGNKVTKG	(3)
Removed	18b	PPSKGQTAIKVTKA	(9)
18–2	18c	PPSKSQTGNKVTKG	This study
18–3	18d	PPSKGQTGNKVTKA	This study
18–4	18e	PPSKGQTGNKVIKG	GenBank AF162345
18–5	18f	PPSKGQVGNKVTKG	(6)
18–6	18g,32	QLSKGQVGNKVTKG	This study
18–7	18h,32a	QPSKGQVGNKVTKG	(8)
18–8	-	PPSKGQTGNKVTNG	GenBank X81111
18–9	-	PPPKDQTGNKVTKG	WS
18–10	-	PPSEGQTGNTVTKA	WS
19	19	PPSKSQPQVKVTKA	(1)
19–1	19a	PPSKSQSQVKVTKA	(10)

19–2	19b	PPSKSQLQVKVTKA	GenBank Z14291
Removed	19c	PASKSQPQVKVTKA	(4)
19–3	19d	PRSKSQPQVKVTKA	(11)
19–4	19e	PPSNSQPQVKVTKA	(11)
19–5	19f	PLSKSQPQVKVTKA	(11)
19–6	19g	PPLKSQPQVKVTKA	(6)
19–7	19h	PSSKSQPQVKVTKA	(6)
19–8	19i	PPPKSQPQVKVTKA	WS
19–9	19j	PPSKSQPQVKVTQVKVTKA	WS
19–10	19k	PHSKSQPQVKVTKA	WS
19–11	-	PPSRSQPQVKVTKA	(12)
19–12	-	PSSKSQSQVKVTKA	WS
19–13	-	PPSKSQTQVKVTKA	WS
19–14	-	PPSKSQHQVKVTKA	WS
20	20	QPQTANTQQGGKVKVTKA	(3)
21	21	QPQVTNGVQGNQVKVTKA	(3)
21–1	21a	QPNGVQGNQVKVTKA	This study
21–2	21b	QPQATNGVQGGQQGNQVKVTKA	This study
21–3	21c	QPQVTKGVQGNQVKVTKA	WS
21–4	21d	QPQVPNGVQGNQVKVTKA	WS
21–5	21e	QPQVPNSVQGNQVKVTKA	WS
21–6	-	QPQATNGVQGGRQGNQVTVTKA	(8)
21–7	-	QLQVTNGVQGNQVKVTKA	WS
22	22	QPSKAQGQTNNQVKVTKA	(1)
22–1	22a	QPSRTQGQTSNQVKVTKA	(8,13)
22–2	22b	QPSRTQAQTSNQVKVTKA	This study
22–3	22c	QPSKAKGQTNNQVKVTKA	WS
22–4	22d	QLSKAQGQTNNQVKVTKA	WS
22–5	-	QPSKAQGQTNNQVKVTKR	WS
Removed	29	PAPKYSTTQVTKA	(4)
31	31	PPSSNQGKNQAQTGNTVTKA	This study

[a]Sequences that have been removed were the result of sequencing errors in the original research. WS, Web site submission.

Appendix References

1. Maiden MCJ, Suker J, McKenna AJ, Bygraves J, Feavers IM. Comparison of the class 1 outer membrane proteins of eight serological reference strains of *Neisseria meningitidis.* Mol Microbiol 1991;5:727–36.
2. Van der Ley P, Heckels JE, Virji M, Hoogerhout P, Poolman JT. Topology of outer membrane porins in pathogenic *Neisseria* spp. Infect Immun 1991;59:2963–71.
3. Suker J, Feavers IM, Achtman M, Morelli G, Wang J-F, Maiden MCJ. The *porA* gene in serogroup A meningococci: evolutionary stability and mechanism of genetic variation. Mol Microbiol 1994;12:253–65.
4. Brooks JL, Fallon RJ, Heckels JE. Sequence variation in class 1 outer membrane protein in *Neisseria meningitidis* isolated from patients with meningococcal infection and close household contacts. FEMS Microbiol Lett 1995;128:145–50.

5. Arhin FF, Moreau F, Coulton J, Mills EL. Sequencing of *por*A from clinical isolates of *Neisseria meningitidis* defines a subtyping scheme and its genetic regulation. Can J Microbiol 1998;44:56–63.
6. Sacchi CT, Whitney AM, Popovic T, Beall DS, Reeves MW, Plikaytis BD, et al. Diversity and prevalence of PorA types in *Neisseria meningitidis* serogroup B in the United States, 1992-1998. J Infect Dis 2000;182:1169–76.
7. McGuiness B, Barlow AK, Clarke IN, Farley JE, Anilionis A, Poolman JT, et al. Deduced amino acid sequences of class 1 protein (PorA) from three strains of *Neisseria meningitidis.* J Exp Med 1990;171:1871–82.
8. Sacchi CT, Lemos APS, Brandt ME, Whitney AM, Melles CEA, Solari CA, et al. Proposed standardisation of *Neisseria meningitidis* PorA variable region typing nomenclature. Clin Diagn Lab Immunol 1998;5:845–55.
9. McGuinness BT, Lambden PR, Heckels JE. Class 1 outer membrane protein of *Neisseria meningitidis*: epitope analysis of the antigenic diversity between strains, implications for subtype definition and molecular epidemiology. Mol Microbiol 1993;7:505–14.
10. Feavers IM, Fox AJ, Gray S, Jones DM, Maiden MCJ. Antigenic diversity of meningococcal outer membrane protein PorA has implications for epidemiological analysis and vaccine design. Clin Diagn Lab Immunol 1996;3:444–50.
11. Wedege E, Caugant DA, Musacchio A, Saunders NB, Zollinger WD. Redesignation of a purported P1.15 subtype-specific meningococcal monoclonal antibody as a P1.19-specific reagent. Clin Diagn Lab Immunol 1999;6:639–42.
12. Sacchi CT, Lemos AP, Popovic T, Cassio de Morais J, Whitney AM, et al. Serosubtypes and PorA types of *Neisseria meningitidis* serogroup B isolated in Brazil during 1997–1998: overview and implications for vaccine development. J Clin Microbiol 2001;39:2897–903.
13. Maiden MCJ, Bygraves JA, McCarvil J, Feavers IM. Identification of meningococcal serosubtypes by polymerase chain reaction. J Clin Microbiol 1992;30:2835–41.
14. Bart A, Dankert J, van der Ende A. Antigenic variation of the class I outer membrane protein in hyperendemic *Neisseria meningitidis* strains in the Netherlands. Infect Immun 1999;67:3842–6.
15. Suker J, Feavers IM, Maiden MCJ. Monoclonal antibody recognition of members of the meningococcal P1.10 variable region family: implications for serological typing and vaccine design. Microbiology 1996;142:63–9.
16. Wedege E, Kolberg J, Delvig A, Hoiby EA, Holten E, Rosenqvist E, et al. Emergence of a new virulent clone within the electrophoretic type 5 complex of serogroup B meningococci in Norway. Clin Diagn Lab Immunol 1995;2:314–21.
17. Saunders NB, Brandt BL, Warren RL, Hansen BD, Zollinger WD. Immunological and molecular characterization of three variant subtype P1.14 strains of *Neisseria meningitidis.* Infect Immun 1998;66:3218–22.
18. Feavers IM, Heath AB, Bygraves JA, Maiden MCJ. Role of horizontal genetic exchange in the antigenic variation of the class 1 outer membrane protein of *Neisseria meningitidis.* Mol Microbiol 1992;6:489–95.
