# Supplementary material for: PorA Variable Regions of Neisseria meningitidis
Source: Emerg Infect Dis. 2004 Apr;10(4):674–8. doi: 10.3201/eid1004.030247 (PMC3323080; doi:10.3201/eid1004.030247)
Supplement: Appendix Table 2 — VR2 sequence nomenclaturea [file 03-0247-appT2-s2.pdf]

Appendix Table 2. VR2 sequence nomenclature[a]

Variant	Previous nomenclature	VR2 peptide sequence	Source or reference
1	1	YVAVENGVAKKVA	(1)
1–1	1a	YVAVENGATKKVA	(6)
1–2	1b	YVAVENGVVKKVA	This study
1–3	1c	YVAVENGVAKKVT	WS
2	2	HFVQQTPKSQPTLVP	(1)
-	2a	found to be 2e	(1)
2–1	2b	HFVQQPPKSQPTLVP	(10)
2–2	2c	HFVQQTPQSQPTLVP	(3)
Removed	2d	HFVQETPKSQPTLVP	(4)
2–3	2e	HFVQQPPKSQLTLVP	This study
2–4	2f	HFVQQTPQSRPTLVP	This study
2–5	2g	HFVQQIPQSQPTLVP	WS
2–6	2i	HFVQQTPTLVP	(6)
2–7	-	HFVQQTSKSQPTLVP	WS
2–8	-	HFVQQTTKSQPTLVP	WS
2–9	-	HFVQQTPQSKPTLVP	WS
2–10	-	HFVQQAPQSQSTLVP	WS
2–11	-	HFVLQTPQSQPTLVP	WS
2–12	-	HFVQQIPKSQPTLVP	WS
2–13	-	YFVQQTPQSQPTLVP	GenBank AF239810
2–14	33	HFVQQKLASKPTLVP	WS
2–15	33a	HFVQQKSTSKPTLVP	WS
2–16	33b	HFVQQKPTSKPTLVP	WS
2–17	33c	HFVQQQPTSEPTLVP	WS
2–18	-	HFVQQIPKSQPILVP	WS
2–19	-	HFVQQTSQSQPTLVP	WS
2–20	-	HFVQQTPIVQQTPKSQPTLVP	WS
3	3	TLANGANNTIIRVP	(3)
3–1	3a	TVANGANNTIIRVP	(14)
3–2	3b	TLANGANDTIIRVP	This study
3–3	-	TLANGADNTIIRVP	WS
3–4	-	TPANGANNTIIRVP	WS
3–5	-	TLAKGANNTIIRVP	WS
4	4	HVVVNNKVATHVP	(13)
4–1	4a	HVVVNNNVATHVP	(8)
4–2	4b	HVVVNNKVATHVPAKVATHVP	This study
4–3	4c	HVVVNNKVTTHVP	WS
9	9	YVDEQSKYHA	(1)
9–1	9a	YVDSKYHA	GenBank AF148643
9–2	-	YVGEQSKYHA	WS
9–3	-	YVDEQSKDHA	WS
9–4	-	YVDKQSKYHA	WS
9–5	-	YVDEQSEYHA	WS
10	10	HFVQNKQNQRPTLVP	(3)
10–1	10a	HFVQNKQNQPPTLVP	(10,15)

10–2	10b	HFVQDKKGQPPTLVP	(10,15)
10–3	10c	HFVQNKQNQQPTLVP	(10,15)
10–4	10d	HFVQNKQNKQNQPPTLVP	(10,15)
10–5	10e	HFVQNKQSQRPTLVP	(3)
10–6	10f	HFVQNKQNQQNQQNQPPTLVP	(10,15)
10–7	10g	HFVQNKQNKPPTLVP	(10,15)
Removed	10h		(4)
Removed	10i		(4)
Removed	10j		(4)
10–8	10k	HFVQNKQNQQNQPPTLVP	This study
10–9	10l	HFVQNKQNKQNQLPTLVP	This study
10–10	10m	HFVQNKQNKQNKQNQPPTLVP	This study
10–11	10n	HFVQNKQNQRSTLVP	This study
10–12	10o	HFVQNKQNQLPTLVP	GenBank AF182278
10–13	10p	HFVQNKQNKKNQPPTLVP	WS
10–14	10q	HFVQNKQHQPPTLVP	WS
10–15	10r	HFVQNKQNQPSTLVP	WS
10–16	10s	HFVQNKQNQWSTLVP	WS
10–17	10t	HFVQNKQNQTPTLVP	WS
10–18	10u	HFVQNKQSQPPTLVP	WS
10–19	10w	HFVQNKQNKQKQPPTLVP	(6)
10–20	10i,10v	HFVQNKQNQWLTLVP	GenBank AF162346
10–21	10x	HFVPDKKGQPPTLVP	(6)
10–22	10y	HFVQNKQNKQNQQNQPPTLVP	WS
10–23	-	HFVQNKQNQWPTLVP	WS
10–24	-	HFVKNKQNQRPTLVP	(6)
10–25	-	HFVQDKKGQP	WS
13	13	YWTTVNTGSATTTTTFVP	(16)
13–1	13a	YWTTVNTGSATTTTFVP	(3)
13–2	13b	YWTTVNTGSATTTFVP	(16)
13–3	13c	YWTTVNTGSATITTFVP	(10)
13–4	13d	YYTTVTQGSATTTTFVP	(10)
13–5	13e	YWTTVNTGSATTTTTTTTTFVP	(16)
13–6	13f	YWTTVNTGSATTTTTTTTFVP	(5)
13–7	13g	YWTTVNTGSATTTTTTTFVP	(5)
13–8	13h	YWITVNTGSATTTTFVP	(16)
13–9	13i	YWTTVNTGSATTFVP	This study
13–10	13j	YWTTVNTGSVTTTTFVP	WS
13–11	13k	YWTTVNTGSAATTTTFVP	This study
13–12	-	YWTAVNAGSATTTFVP	WS
14	14	YVDEKKMVHA	(13)
14–1	14a	YVDEKKKMVHA	(8)
14–2	14b	YVDEKKKVHA	(17)
14–3	14c	YVDEKNMVHA	(17)
14–4	14d	YVDENKMVHA	(11)
14–5	14e	YVDKEQVSHA	This study
14–6	14f	YVDEKQVSHA	This study
14–7	14g	YVDETKMVHA	WS
14–8	-	YVDEKRMVHA	WS
14–9	-	YVDAKKMVHA	WS
15	15	HYTRQNNADVFVP	(1)
15–1	15a	HYTRQNNTDVFVP	(7)

15–2	15c	HYTRQNNNNTDVFVP	This study
15–3	15d	HYTRPNNTDVFVP	(11)
15–4	15e	HYNTRQNNADVFVP	WS
15–5	15f	HYTRQNSADVFVP	This study
15–6	15g	HYTRQNYADVFVP	WS
15–7	15h	HYTRQNNANVFVP	(6)
15–8	-	HYTRQNNAGVFVP	(12)
15–9	-	HYTRQNNTRQNNADVFVP	(12)
15–10	-	HYTGQNNADVFVP	(12)
15–11	15b	HYTRQNNIDVFVP	(10)
15–12	-	HYNTRQNNIDVFVP	WS
15–13	-	HYTRQNNQNNIDVFVP	WS
15–14	-	HYTNTRQNNIDVFVP	WS
15–15	-	HYTRQSNTDVFVP	WS
15–16	-	HYTRQNNADFVP	WS
16	16	YYTKDTNNNLTLVP	(1)
16–1	16w	YYTKGKNNALTLVP	WS
16–2	16b	YYTKNTNNNLTLVP	(18)
16–3	16c	YYTKDKNDNLTLVP	(5)
16–4	16d	YYTKDKNDKLTLVP	(5)
16–5	16e	YYTKDTNNNNNLTLVP	(5)
16–6	16f	YYTKHTNNNLTLVP	This study
16–7	16g	YYTKDTNTKDTNNNLTLVP	This study
16–8	16h	YYTKDKNNALTLVP	(6)
16–9	16i	YYTKDTNDLTLVP	WS
16–10	16j	YYTNNNLTLVP	This study
16–11	16k	YYTTDTNNNLTLVP	This study
16–12	16l	YYTKDTNDNLTLVP	This study
16–13	16m	YYTEDTNNNLTLVP	WS
16–14	16n	YYTKDTNTNLTLVP	WS
16–15	16p	YYNTKDTNNNLTLVP	This study
16–16	16q	YYTKDTNNNPTLVP	This study
16–17	16r	YYTKDTNNTNNNLTLVP	(6)
16–18	16s	YYTKDTNTNNNLTLVP	GenBank AF143744
16–19	16t	YYTKDTNNNLTHTKDTNNNLTLVP	(6)
16–20	16u	KDTNNNLTLVP	(6)
16–21	16v	YYTKDTKNNLTLVP	(6)
16–22	-	YYTKDTNNILTLVP	WS
16–23	-	YYTKDNKNDNLTLVP	(6)
16–24	-	YYTKVENDNLTLVP	WS
16–25	-	YYTKDTNNNLNLTLVP	WS
16–26	-	YYTNTNNNLTLVP	WS
16–27	-	YYTKDTNNNLTLVS	WS
23	23	HWNTVYNTNGTTTTFVP	(3)
23–1	23a	HWNTVYNTNGTTTTTTTFVP	(6)
23–2	-	HWNTVYNTNGTTTTTFVP	WS
Removed	24	TLANVANTNIGVP	(13)
25	25	TYTVDSSGVVTPVP	(1)
25–1	25a	TYTVDSSGVFTPVP	This study
25–2	25b	TYTEGSSGVFTPVP	WS
25–3	25c	TYTVDSSGVVTPLP	WS
25–4	25d	TYTVGSRDVVTPVP	GenBank AF162345

25–5	25e	TYTVDSSNVVTPVP	GenBank AF157834
25–6	25f	TYTVDSGVVTPVP	(6)
25–7	25g	YTVDSSGVVTPVP	WS
25–8	-	TYTVDSSGVP	WS
25–9	-	TYTVDNSSVVTPVP	WS
26	26	HFVADSQGKITRVP	(10)
26–1	26a	HFVADSQGEITRVP	GenBank AF146084
26–2	-	YFTADPNDQNKITRVP	WS
28	28	YYYTTATNSSTSTTFVP	(10)
30	30	HYTTVYNATTTTTTFVP	WS
30–1	30a	HYTTVYNATTTTTFVP	This study
30–2	30b	HYTTVYNATTTTTTTFVP	(6)
30–3	30c	HYTTVYNATTTTTTTTFVP	This study
30–4	30d	HYTTVYNATTTTTTTTTFVP	WS
30–5	30e	HYTTVYNATTTTTTTTTTFVP	WS
34	34	YVDDQGKVKGP	(6)
34–1	-	YVDDQKVKGP	WS
35	-	TFTLESNQMKPVP	WS

Appendix References

1. Maiden MCJ, Suker J, McKenna AJ, Bygraves J, Feavers IM. Comparison of the class 1 outer membrane proteins of eight serological reference strains of *Neisseria meningitidis*. Mol Microbiol 1991;5:727–36.
2. Van der Ley P, Heckels JE, Virji M, Hoogerhout P, Poolman JT. Topology of outer membrane porins in pathogenic *Neisseria* spp. Infect Immun 1991;59:2963–71.
3. Suker J, Feavers IM, Achtman M, Morelli G, Wang J-F, Maiden MCJ. The *porA* gene in serogroup A meningococci: evolutionary stability and mechanism of genetic variation. Mol Microbiol 1994;12:253–65.
4. Brooks JL, Fallon RJ, Heckels JE. Sequence variation in class 1 outer membrane protein in *Neisseria meningitidis* isolated from patients with meningococcal infection and close household contacts. FEMS Microbiol Lett 1995;128:145–50.
5. Arhin FF, Moreau F, Coulton J, Mills EL. Sequencing of *por*A from clinical isolates of *Neisseria meningitidis* defines a subtyping scheme and its genetic regulation. Can J Microbiol 1998;44:56–63.
6. Sacchi CT, Whitney AM, Popovic T, Beall DS, Reeves MW, Plikaytis BD, et al. Diversity and prevalence of PorA types in *Neisseria meningitidis* serogroup B in the United States, 1992-1998. J Infect Dis 2000;182:1169–76.
7. McGuiness B, Barlow AK, Clarke IN, Farley JE, Anilionis A, Poolman JT, et al. Deduced amino acid sequences of class 1 protein (PorA) from three strains of *Neisseria meningitidis*. J Exp Med 1990;171:1871–82.
8. Sacchi CT, Lemos APS, Brandt ME, Whitney AM, Melles CEA, Solari CA, et al. Proposed standardisation of *Neisseria meningitidis* PorA variable region typing nomenclature. Clin Diagn Lab Immunol 1998;5:845–55.

9. McGuinness BT, Lambden PR, Heckels JE. Class 1 outer membrane protein of *Neisseria meningitidis*: epitope analysis of the antigenic diversity between strains, implications for subtype definition and molecular epidemiology. Mol Microbiol 1993;7:505–14.
10. Feavers IM, Fox AJ, Gray S, Jones DM, Maiden MCJ. Antigenic diversity of meningococcal outer membrane protein PorA has implications for epidemiological analysis and vaccine design. Clin Diagn Lab Immunol 1996;3:444–50.
11. Wedege E, Caugant DA, Musacchio A, Saunders NB, Zollinger WD. Redesignation of a purported P1.15 subtype-specific meningococcal monoclonal antibody as a P1.19-specific reagent. Clin Diagn Lab Immunol 1999;6:639–42.
12. Sacchi CT, Lemos AP, Popovic T, Cassio de Morais J, Whitney AM, et al. Serosubtypes and PorA types of *Neisseria meningitidis* serogroup B isolated in Brazil during 1997–1998: overview and implications for vaccine development. J Clin Microbiol 2001;39:2897–903.
13. Maiden MCJ, Bygraves JA, McCarvil J, Feavers IM. Identification of meningococcal serosubtypes by polymerase chain reaction. J Clin Microbiol 1992;30:2835–41.
14. Bart A, Dankert J, van der Ende A. Antigenic variation of the class I outer membrane protein in hyperendemic *Neisseria meningitidis* strains in the Netherlands. Infect Immun 1999;67:3842–6.
15. Suker J, Feavers IM, Maiden MCJ. Monoclonal antibody recognition of members of the meningococcal P1.10 variable region family: implications for serological typing and vaccine design. Microbiology 1996;142:63–9.
16. Wedege E, Kolberg J, Delvig A, Hoiby EA, Holten E, Rosenqvist E, et al. Emergence of a new virulent clone within the electrophoretic type 5 complex of serogroup B meningococci in Norway. Clin Diagn Lab Immunol 1995;2:314–21.
17. Saunders NB, Brandt BL, Warren RL, Hansen BD, Zollinger WD. Immunological and molecular characterization of three variant subtype P1.14 strains of *Neisseria meningitidis*. Infect Immun 1998;66:3218–22.
18. Feavers IM, Heath AB, Bygraves JA, Maiden MCJ. Role of horizontal genetic exchange in the antigenic variation of the class 1 outer membrane protein of *Neisseria meningitidis*. Mol Microbiol 1992;6:489–95.
